# Galectin-3 Performance in Histologic and Cytologic Assessment of Thyroid Nodules: A Systematic Review and Meta-Analysis

**DOI:** 10.3390/ijms18081756

**Published:** 2017-08-11

**Authors:** Pierpaolo Trimboli, Camilla Virili, Francesco Romanelli, Anna Crescenzi, Luca Giovanella

**Affiliations:** 1 Department of Nuclear Medicine and Thyroid Centre, Oncology Institute of Southern Switzerland, Via Ospedale, 6500 Bellinzona, Switzerland; luca.giovanella@eoc.ch; 2 Department of Medico-surgical Sciences and Biotechnologies, Sapienza University of Rome, 04100 Latina, Italy; camillavirili@libero.it; 3 Department of Experimental Medicine, Sapienza University of Rome, 00161 Rome, Italy; francesco.romanelli@uniroma1.it; 4 Pathology Unit, University Hospital Campus Bio Medico, 00128 Rome, Italy; a.crescenzi@unicampus.it

**Keywords:** thyroid cancer, meta-analysis, cytology, Galectin-3, fine needle aspiration (FNAC)

## Abstract

The literature on Galectin-3 (Gal-3) was systematically reviewed to achieve more robust information on its histologic reliability in identifying thyroid cancers and on the concordance between Gal-3 test in histologic and cytologic samples. A computer search of the PubMed and Scopus databases was conducted by combinations of the terms thyroid and Gal-3. Initially, 545 articles were found and, after their critical review, 52 original papers were finally included. They reported 8172 nodules with histologic evaluation of Gal-3, of which 358 with also preoperative FNAC Gal-3 assessment. At histology, Gal-3 sensitivity was 87% (95% confidence intervals [CI] from 86% to 88%), and specificity 87% (95% CI from 86% to 88%); in both cases, we found heterogeneity (I_2_ 85% and 93%, respectively) and significant publication bias (*p* < 0.001). The pooled rate of positive Gal-3 at fine needle aspiration (FNAC) among cancers with histologically proven Gal-3 positivity was 94% (95% CI from 89% to 97%), with neither heterogeneity (I_2_ 14.5%) nor bias (*p* = 0.086). These data show high reliability of Gal-3 for thyroid cancer at histology, while its sensitivity on FNAC samples is lower. The limits of cytologic preparations and interpretation of Gal-3 results have to be solved.

## 1. Introduction

The mammalian galectins are a family of soluble sugar binding proteins characterized by a carbohydrate recognition domain that shows a high affinity for β-galactosides [1]. Among the galectin family, Galectin-3 (Gal-3) is the only chimeric type. It is composed of a proline- and glycin-rich amino-terminal domain fused to a carboxy-terminal carbohydrate recognition domain. This domain recognizes and binds β-galactosides on cell glycoproteins and glycolipids. Gal-3 may be observed in the cytoplasm and in the nucleus as well as extracellular matrix [2]. The expression and distribution of Gal-3 between the nucleus and cytosol changes during cell differentiation and cancer development [3]. This protein is involved in a large number of physiological and pathological processes such as cell proliferation, differentiation, survival, apoptosis, intracellular trafficking and tumor progression [4]. Then, Gal-3 expression was reported in tumors of different organs: in thyroid, liver, stomach, and central nervous system, the protein is up-regulated, whereas, in cancers of the breast, ovary, uterus and prostate, it is down-regulated [5]. Nuclear Gal-3 is involved in various functions such as regulation of gene transcription; within thyroid cells, it modulates gene transcription by the interaction with the nuclear thyroid-specific transcription factor TTF-1 [6]. Gal-3 has received significant attention for its utility as a diagnostic marker for thyroid cancer, being differentially expressed in thyroid carcinoma compared with benign and normal thyroid specimens [7].

In thyroid nodules, Gal-3 expression was demonstrated by immunohistochemistry (IHC) utilizing histologic or cytologic specimens; numerous studies investigated Gal-3 in thyroid malignancy either as a single marker or as a part of a molecular panel. Variability in the reported performances of this marker, however, was frequently seen; a standardized protocol has been proposed and published several years ago by experts in the fields for widespread use of Gal-3 [8]. Recent studies have also demonstrated improved methodological reliability for Gal-3 expression in thyroid fine-needle aspiration (FNAC) material by performing the IHC evaluation on cell-block preparation so avoiding problems related to antigen accessibility in cell smears. The use of cell-block for performing IHC for diagnostic marker is the only recommended by American Thyroid Association (ATA) [9].

Following the above open problems, the actual reliability of Gal-3 in detecting thyroid cancers remains not definitely assessed. Thus, here we aimed to systematically review the literature to achieve more robust information about: (1) the histologic sensitivity and specificity of Gal-3 in the identification of thyroid malignancy; and (2) the concordance between histologic results and preoperative data obtained in FNAC samples. The latter was performed to emphasize the reliability of Gal-3 test in the pre-surgical diagnostic workup. According to these objectives, we designed a very careful selection of papers for meta-analysis searching those paper reporting Gal-3 evaluation in both FNAC and histologic specimens in the same patient.

## 2. Results

### 2.1. Eligible Articles and Description of the Studies

**Figure 1:**
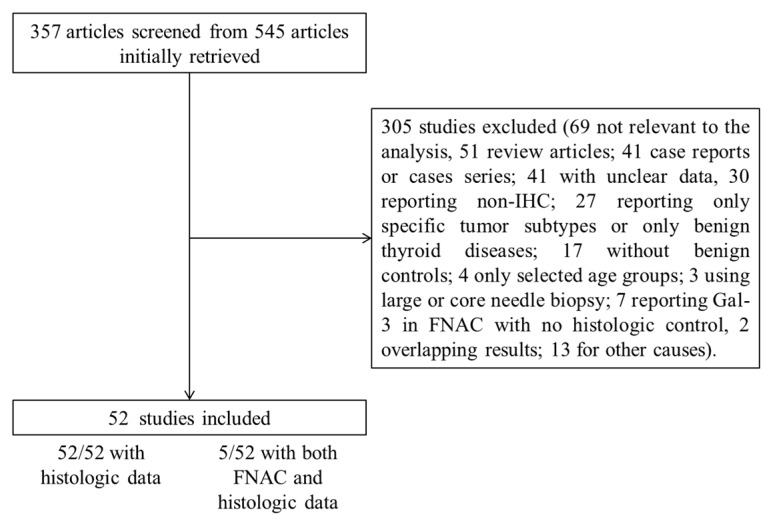
Diagram of flow of search strategy and results.

The comprehensive computer literature search revealed 545 articles. Once duplicates articles were excluded, the papers initially included were 357. Abstracts of these articles were screened and 305 were excluded according to the abovementioned criteria; specifically, 41 were excluded due to unclear data and another seven due to incomplete data on Gal-3 (i.e., Gal-3 performed only in FNAC samples with no correlated data on histologic specimens). Two papers were excluded after contacting authors due to potential overlapping data with other studies. Finally, the systematic review included 52 original articles reporting Gal-3 IHC in histologic samples [10,11,12,13,14,15,16,17,18,19,20,21,22,23,24,25,26,27,28,29,30,31,32,33,34,35,36,37,38,39,40,41,42,43,44,45,46,47,48,49,50,51,52,53,54,55,56,57,58,59,60,61], of which five with both FNAC and histologic examination of Gal-3 in the same series of lesions [13,15,26,38,43]. Figure 1 illustrates the diagram of flow to retrieve the final series of papers.

### 2.2. Qualitative Analysis

The fifty-two papers included [10,11,12,13,14,15,16,17,18,19,20,21,22,23,24,25,26,27,28,29,30,31,32,33,34,35,36,37,38,39,40,41,42,43,44,45,46,47,48,49,50,51,52,53,54,55,56,57,58,59,60,61] were published from 1995 to 2016. Authors were European in 25 cases, while 15 were from Asia, seven from USA, four from Africa, and one from Latin America. All studies had a retrospective design. Overall, more than eight thousand lesions were submitted to Gal-3 test at histology, of which half were malignant. As mentioned above, five papers reported Gal-3 results obtained in the same patients in both FNAC and histology; one of these [38] was excluded from FNAC analysis because Gal-3 was evaluated on conventional smears from FNAC which are not the gold standard for ancillary examinations such as IHC. The remaining four studies [13,15,26,43] described 358 cases, of which 142 cancers and 216 benign nodules. Table 1 summarizes the data from the 52 studies included for the meta-analysis. Notably, the large majority of benign lesions with positivity of Gal-3 were represented by adenomas and thyroiditis, probably because Gal-3 is expressed in the cytosol of thyrocytes and blocks the apoptotic pathway (this condition may be present in these benign lesions).

### 2.3. Quantitative Analysis (Meta-Analysis) of Gal-3 on Histology

**Figure 2:**
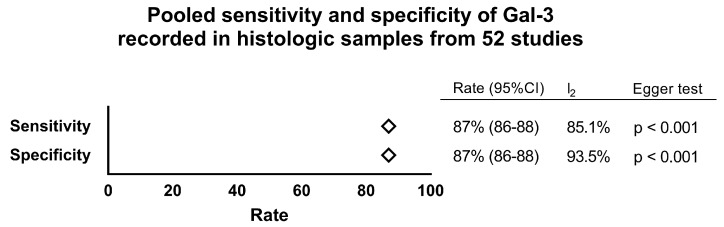
Forest plot of pooled sensitivity and specificity (fixed-effect) of Gal-3 test at histology of 4237 thyroid cancers and 3935 thyroid benign nodules from 52 studies. High heterogeneity (I_2_) and significant publication bias (Egger test) were observed.

The histologic performance of Gal-3 in histologic specimens was evaluated by all 52 studies. The pooled rate of Gal-3 positive results among carcinomas (i.e., Gal-3 sensitivity for thyroid malignancy histology) was 87% (95% CI from 86% to 88%), ranging from 53% to 100%. The series of cancers was heterogeneous (I_2_ 85.1%, 95% CI from 81.7% to 87.8%) and showed significant publication bias (Egger test: −2.53, 95% CI from −3.59 to −1.47, *p* < 0.001). The pooled percentage of benign lesions with negative Gal-3 (i.e., Gal-3 specificity for thyroid benignancy histology) was 87% (95% CI from 86% to 88%), ranging from 20% to 100%. This series was heterogeneous (I_2_ 93.5%, 95% CI from 92.5% to 94.3%) and showed significant publication bias (Egger test: −3.59, 95% CI from −4.85 to −2.32, *p* < 0.001). Figure 2 summarizes the performance of Gal-3 at histology.

In an attempt to avoid the above heterogeneity and risk of bias, we meta-analyzed only the larger series and selected papers with at least 200 cases with histologic follow-up [16,27,30,37,41,44,47,48,55]; nevertheless, heterogeneity and publication bias were still present (sensitivity and specificity were 89% and 92%, respectively). On the other hand, when we evaluated a subseries of papers using biotin-free method for Gal-3 [18,21,26,27,28,34,35,37,40,43,47,50,54,57,59], interestingly we found absence of publication bias in the calculation of sensitivity (86% sensitivity, I_2_ 88.3%, 95% CI from 82.7% to 91.4%, Egger test: −2.9, 95% CI from −6.17 to 0.37, *p* = 0.078; 84% specificity, I_2_ 91.8%, 95% CI from 88.7% to 93.8%, Egger test: −4,09, 95% CI from −7.22 to −0.97, *p* = 0.014).

### 2.4. Quantitative Analysis (Meta-Analysis) of Gal-3 on FNAC, and Concordance of Cytologic Results with Histologic Ones

The reliability of Gal-3 when performed in cell-block from FNAC was investigated and reported in four papers [13,15,26,43].

Initially, we analyzed the histologic results in this subgroup: the pooled histologic sensitivity of Gal-3 was 96% (95% CI from 92% to 99%), and neither heterogeneity (I_2_ 19.1%, 95% CI from 0% to 73.6%) nor significant bias (Egger test: −0.55, 95% CI from −6.55 to 5.44, *p* = 0.73) was found; in addition, histologic specificity of Gal-3 was 89% (95% CI from 85% to 93%), and there was neither heterogeneity (I_2_ 0%, 95% CI from 0% to 67.9%) nor significant publication bias (Egger test: −0.6, 95% CI from −13.59 to 12.38, *p* = 0.86).

Then, we evaluated the pooled rate of preoperative Gal-3 positivity between cancers (i.e., Gal-3 sensitivity for thyroid malignancy at FNAC): the sensitivity of Gal-3 was 90% (95% CI from 85% to 94%), ranging from 80% to 94%; the series of cancers was not heterogeneous (I_2_ 0%, 95% CI from 0% to 67.9%) and showed no significant publication bias (Egger test: −0.87 (95% CI from −4.29 to 2.55, *p* = 0.389).

**Figure 3:**
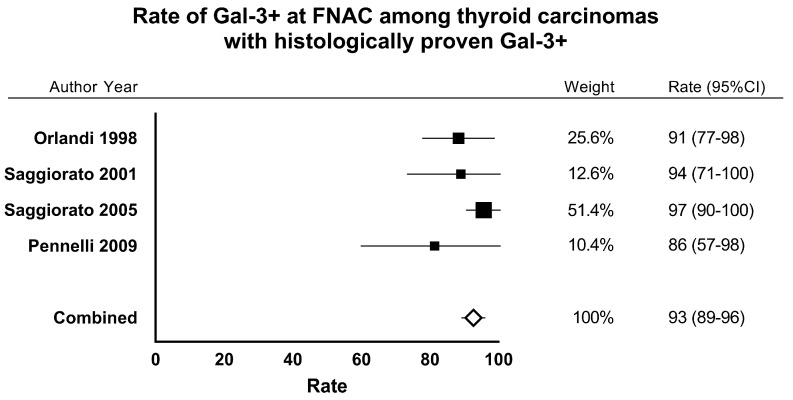
Forest plot of meta-analysis (fixed-effect) of proportion of FNAC samples (cell block preparation) with positivity of Gal-3 test among thyroid cancers with histologically proven positivity of Gal-3.

Finally, we calculated the concordance between Gal-3 results at histology with Gal-3 at FNAC. The pooled rate of positive Gal-3 at FNAC within the series of cancers with positive Gal-3 test at histology was 94% (95% CI from 89% to 97%); the series was not heterogeneous (I_2_ 14.5%, 95% CI from 0% to 72.3%) and there was no publication bias (Egger test: −1.25 (95% CI from −2.95 to 0.44, *p* = 0.086) (Figure 3). Perfect concordance (100%) was found between negative Gal-3 results at FNAC (*n* = 216) and histology (*n* = 216).

## 3. Discussion

The major result of the present meta-analysis is that Gal-3 IHC is positive in 87% of thyroid cancers, as proven at analysis of histologic findings. This is pivotal information because it demonstrates that many thyroid carcinomas have over-expression of this marker. The weakness of this result is that we observed very relevant heterogeneity between the studies and significant publication bias. However, it is reasonable to consider this percentage as quite reliable due to the short 95% CI of sensitivity. In addition, 87% of benign lesions are proven to be Gal-3 negative at histology. High heterogeneity, significant publication bias, and short 95% CI were also recorded in specificity. Interestingly, we found a significant improvement of results of sensitivity of Gal-3 when we meta-analyzed only those papers using a biotin-free method. This indirectly demonstrates the Gal-3 has to be evaluated by biotin-free approach. An interesting study on Gal-3 in preoperative samples and definitive histologic specimens was published by Bartolazzi et al. [7]. Unfortunately, this study was excluded from our meta-analysis due to high risk of overlapping data. In that study, 465 nodules were tested for Gal-3 (biotin-free method), 134 of these were positive and 101/134 were carcinomas (the remaining false positive cases were largely represented by benign neoplasia such as adenomas). Table 2 reports the most significant results from this study.

Interestingly, this study [7] is the most relevant in the literature and was conducted according to a specific protocol [8]. Thus, our present study strongly corroborates those findings and demonstrates, with evidence-based data, that the vast majority of thyroid malignancy is Gal-3 positive.

The most relevant application of Gal-3 is obviously in the assessment of thyroid nodules, especially when the conventional cytologic report is non-conclusive/indeterminate. The present meta-analysis shows that Gal-3 test on FNAC samples (cell-block preparation) has a sensitivity lower than that recorded at histology. We have to underline that some studies of this subgroup were published by the same authors; there were no overlapping data (we contacted the authors), but it might represent a potential risk of bias. However, many problems might affect these data. In fact, among the different methodologies used for Gal-3 evaluation in thyroid specimens, there is great technical variation in antibody clones and characteristics, antibody dilution, antigen retrieval methods and biotin free protocols for reaction visualization. Moreover, relevant differences exist in staining interpretation: discordance among pathologists occurs for marker localization (nuclear, cytoplasm and cell membrane) and there is still lack of fixed cutoff for positive classification (i.e., qualitative [weak, moderate, and intense] and quantitative [5%, 10%, 50%, etc.]) [62]. The scoring criterion is a crucial step to improve the reliability of molecular markers for clinical use and then carry out their sensitivity and specificity. As for other markers used as oncological diagnostic, prognostic and predictive tools, Gal-3 strongly needs for a standardized protocol and laboratory validation to establish its actual utility and reliability. Importantly, the laboratory validation of Gal-3 test-method for thyroid cancer diagnosis has already been extensively performed, and two papers have adopted it [7,16]. This strongly encourages using Gal-3 protocol [8]. Recent studies about other markers reported improved accuracy in protein expression analysis by the use of RNA in situ hybridization especially when there is no suitable antibody or the target molecule is a secreted protein [63]. Moreover, the RNAscope 2.0 assay system (Advanced Cell Diagnostics, Hayward, CA, USA) is a relatively new technology accomplished with recommended scoring that allows reliable mRNA assessment on a glass slide [64]. After studies in thyroid pathology, mRNA in situ evaluation approach could integrate or replace IHC for Gal-3 for its use in clinical practice. As a potential weakness of this evaluation, it should be underlined that a scantly (i.e., undetectable at IHC) Gal-3 expression is normal in nucleosome of normal thyroid cell because there Gal-3 plays a physiological function as transcriptional regulator. Thus, mRNA in situ evaluation might carry a risk of potential false positive results. As mentioned above, one study evaluated Gal-3 in FNAC smears and was excluded from meta-analysis of cytologic reliability of Gal-3 [38]; however, regardless this cytologic preparation is not the gold standard for IHC, the authors reported good sensitivity.

A discussion for clinical practice may be addressed. After defining the above issues of the preoperative Gal-3 use, we could have a significant improvement in the management of those patients with indeterminate FNAC reports; in fact, thyroid cancers are not frequent in this class of patients (i.e., up to 25%) and the prognosis of malignant lesions with this preoperative assessment is really good [65,66]. This encourages reducing diagnostic thyroidectomy in patients with indeterminate FNAC and negative Gal-3 result [67]. The use of Gal-3 test should have a favorable impact on the costs for management of our patients especially when compared with other molecular testing [68], even if this has to be proven in a prospective study, if possible.

Finally, other preoperative uses of Gal-3 test were reported in literature. Specifically, high reliability of Gal-3 was recorded in microhistologic specimens from core needle biopsy [69,70,71]; the latter results have to be confirmed but this technique has not been diffused. In addition, Gal-3 was investigated as a serum marker; unfortunately a few studies on small series have been reported in this topic and no significant difference between cancers and benign lesion were found. These findings are probably due to the sub- intra-cellular localization of Gal-3 and the lack of its secretory pathway. To date, the role of serum Gal-3 is excluded [40,45,72,73,74]. Two papers investigated an imaging Gal-3 approach [75,76], but until now no data on application of these radiolabelled antibodies in humans exist. As these studies come from the same group, an independent validation should be desirable before proceeding with phase I clinical studies. Notably, other widely employed nuclear medicine procedures (i.e., 99mTc-sestaMIBI scan and 18FDG-PET/CT) already show good accuracy in discriminating cytologically indeterminate nodules [77,78,79,80,81].

In conclusion, the present meta-analysis demonstrates that a high rate of thyroid cancers has Gal-3 over-expression at histology and elevated percentage of thyroid benign nodules has negative Gal-3 test. However, high heterogeneity and significant publication bias were evident. In addition, the sensitivity of Gal-3 test on FNAC samples is lower than that recorded at histology, prompting to solve limits of cytologic preparations and interpretation of results.

## 4. Materials and Methods

### 4.1. Search Strategy

Initially, we searched studies investigating the role of Galectin-3 in diagnosing thyroid nodules. A comprehensive computer literature search of the PubMed/MEDLINE and Scopus databases was conducted to find published articles on this topic. The search algorithm was based on the combinations of the terms “thyroid” and “galectin” (i.e., “thyro*” AND “galect*” on PubMed/MEDLINE: by this search we could find all combinations of terms beginning with thyro and those beginning with galect). A beginning date limit was not used, and the search was updated until 14 May 2017. The research was restricted to English language studies. To identify additional studies and expand our search, references lists of the retrieved articles could be also screened. Three investigators (Pierpaolo Trimboli, Camilla Virili, and Francesco Romanelli) independently searched articles applying the above strategy.

### 4.2. Study Selection

Original articles reporting experience on the use of Gal-3 in humans were eligible for inclusion. In particular, we selected studies encompassing analysis of Gal-3 IHC on malignant and benign thyroid lesions; we also selected papers about Gal-3 usefulness in preoperatively made FNAC in ameliorating cytologic diagnosis of thyroid tumors. Specifically, only studies using formalin fixed and paraffin embedded histologic samples and cell-block cytologic samples were considered as eligible for the final series to be meta-analyzed. Studies using non-IHC technique for Gal-3 evaluation, review articles, case reports, cases series, articles with unclear data, and series with overlapping results were also excluded. The same three authors (PT, CV, FR) independently screened titles and abstracts of the retrieved articles according to the above criteria, reviewed the full-texts, and selected articles for their inclusion.

### 4.3. Data Extraction

For each included study, information was extracted concerning study data: authors, year of publication, journal, study aim, study design, main results, type of Gal-3 evaluation (i.e., cytologic and/or histologic specimens, conventional or cell-block preparation) and country of patient’s origin. Number, gender, and age of patients enrolled in the studies were also collected, when available.

### 4.4. Statistical Analysis

For statistical pooling of the data, the fixed-effects model was used. Pooled data were presented with 95% confidence intervals (95% CI) and displayed using a forest plot. I-square index was used to quantify the heterogeneity among the studies, and a significant heterogeneity was defined as an I-square value > 50%. Egger test was carried out to evaluate the possible presence of a significant publication bias. Statistical significance was set at *p* < 0.01. All analyses were performed using the StatsDirect statistical software version (StatsDirect Ltd., Altrincham, UK).

## Figures and Tables

**Table 1:** Summary of results of the 52 studies included for the meta-analysis.

Samples	Total Lesions	Carcinomas	Carcinomas with Gal-3+ (%)	Benign Lesions	Benign Lesions with Gal-3− (%)
Histologic data	8172	4237	3654 (86%)	3935	3341 (85%)
Cytologic data (cell-block preparation from FNAC samples)	358	142	129 (91%)	216	194 (90%)

**Table 2:** Results obtained in the multicenter study by Bartolazzi et al. [7]: Galectin-3 immunocytochemical evaluation on 465 follicular thyroid proliferations compared to the final histology.

Samples	Malignant	Benign	Borderline	Positive Predictive Value
Galectin-3 positive	101	22	11	82%
Galectin-3 negative	280	29	22	91%
